# Microarray Analysis of Human Monocytes Infected with *Francisella tularensis* Identifies New Targets of Host Response Subversion

**DOI:** 10.1371/journal.pone.0002924

**Published:** 2008-08-13

**Authors:** Jonathan P. Butchar, Thomas J. Cremer, Corey D. Clay, Mikhail A. Gavrilin, Mark D. Wewers, Clay B. Marsh, Larry S. Schlesinger, Susheela Tridandapani

**Affiliations:** 1 Division of Pulmonary and Critical Care, Department of Internal Medicine, The Ohio State University, Columbus, Ohio, United States of America; 2 Molecular, Cellular and Developmental Biology Program, The Ohio State University, Columbus, Ohio, United States of America; 3 Center for Microbial Interface Biology, The Ohio State University, Columbus, Ohio, United States of America; Federal University of São Paulo, Brazil

## Abstract

*Francisella tularensis* is a gram-negative facultative bacterium that causes the disease tularemia, even upon exposure to low numbers of bacteria. One critical characteristic of *Francisella* is its ability to dampen or subvert the host immune response. In order to help understand the mechanisms by which this occurs, we performed Affymetrix microarray analysis on transcripts from blood monocytes infected with the virulent Type A Schu S4 strain. Results showed that expression of several host response genes were reduced such as those associated with interferon signaling, Toll-like receptor signaling, autophagy and phagocytosis. When compared to microarrays from monocytes infected with the less virulent *F. tularensis subsp. novicida*, we found qualitative differences and also a general pattern of quantitatively reduced pro-inflammatory signaling pathway genes in the Schu S4 strain. Notably, the PI3K / Akt1 pathway appeared specifically down-regulated following Schu S4 infection and a concomitantly lower cytokine response was observed. This study identifies several new factors potentially important in host cell subversion by the virulent Type A *F. tularensis* that may serve as novel targets for drug discovery.

## Introduction

*Francisella tularensis* is a gram-negative facultative bacterium that is phagocytosed by mononuclear phagocytes [1] and polymorphonuclear leukocytes in the presence of serum [1], [2], and also enters epithelial cells [3]. *F. tularensis*, similar to pathogens like *L. Monocytogenes* [4] is known as a stealth pathogen as it escapes the phagosome and replicates inside the host cell cytosol [5], eventually leading to host cell death [6], [7]. How this bacterium is able to evade normal phagolysosomal fusion and continue to survive and proliferate is of great interest.

To help shed light on how *F. tularensis* survives successfully inside the host cell, we previously performed Affymetrix oligonucleotide microarray analysis to examine genome wide transcriptional responses of human monocytes to the lesser virulent *F. novicida subsp novicida* strain. Results showed that several pro-inflammatory mediators were up-regulated following infection [8], many of which had been previously described such as Interferon-γ (IFNγ) [9], [10], [11], [12], Interleukin-2 (IL-2) [9], [10], Interleukin-12 (IL-12) [12], Interleukin-23 (IL-23) [8] and Interleukin-1β (IL-1β) [13], [14]. However, it has been shown that after an initial pro-inflammatory period, there is a marked down-regulation in cytokine and signaling responses that depend on phagosomal escape [15].

Here, we have performed a microarray analysis of human peripheral blood monocytes after infection with the highly virulent Type A *F. tularensis subsp. tularensis* Schu S4 strain. We found down-regulation of genes in several general immune response pathways such as phagocytosis, autophagy, IFNγ signaling and Toll-like Receptor (TLR) signaling. We have extended these studies to show that, this down-regulation of TLR pathway genes results in dampening of the monocyte response to subsequent stimulation with TLR ligands. Further, the PI3K / Akt pathway, shown to be critical for optimal cytokine responses [16], [17], was preferentially down-regulated in *F. tularensis subsp. tularensis* Schu S4 when compared to *F. tularensis subsp. novicida* infection with a concomitant down-regulation in cytokine production. This large-scale view of the host response to *Francisella* shows a pattern of dampened monocyte responses in several pro-inflammatory pathways following infection by a Type A prototype strain which provides greater insight into mechanisms underlying its highly virulent nature. The sub-optimal response by elements of these pathways uncovers them as potential therapeutic targets.

## Materials and Methods

### Cells and Reagents

THP-1 cells (ATCC) and human peripheral blood monocytes were cultured in RPMI 1640 containing 10% heat-inactivated FBS, 2 mM L-glutamine and 50 units / ml penicillin-streptomycin. Antibodies against Akt1, MyD88 and MKP1 were from Santa Cruz Biotechnologies (Santa Cruz, CA). PE-Cy7-conjugated anti-CD14 and isotype control were from BD Pharmingen (San Diego, CA). Anti-IFNγRI was from R & D Systems (Minneapolis, MN) and the FITC-conjugated secondary was from Caltag / Invitrogen (Carlsbad, CA). Purified *E. coli* (strain 0127∶B8) LPS was from Difco (Detroit, MI) and Pam_3_Cys-Ser-(Lys)_4_ (Pam3CSK) was from Calbiochem / EMD (San Diego, CA). Gentamicin Reagent Solution was from Invitrogen.

### Peripheral blood monocyte isolation

Human peripheral blood monocytes were isolated using either CD14 positive or negative selection by centrifugation through a Ficoll gradient followed by MACS (Miltenyi Biotec, Auburn, CA) in accordance with manufacturer instructions. Flow cytometry using CD14 antibody showed a minimum of 96% purity for each sample.

### Infections

Monocytes were infected at a multiplicity of infection (MOI) of 100, with 5 million monocytes and 500 million *F. tularensis novicida* or *F. tularensis tularensis* Schu S4, then incubated in 1 ml RPMI with 10% FBS for 24 hours at 37° C. Bacteria were grown overnight (approximately 18 hours) on chocolate II agar (Becton Dickinson) at 37°C prior to use. Infections with Schu S4 were performed in a BSL3 facility at The Ohio State University as previously described [8].

### Microarray analysis

RNA was extracted using Trizol (Invitrogen) and subsequent labeling and hybridization to Affymetrix hgu133plus2 chips was performed at The Ohio State University Comprehensive Cancer Center microarray facility. Resulting data files (.CEL) were pooled with earlier .CEL files [8] and all .CEL files were then preprocessed and analyzed using R and BioConductor [18], [19]. Expression values were calculated using the “gcrma” package and both the statistical analyses for differential expression and creation of the Venn diagram were done using the “limma” package [20]. Expression values and analysis results were stored in a PostgreSQL database (http://www.postgresql.org). The data discussed in this publication have been deposited in NCBI's Gene Expression Omnibus [21] and are accessible through GEO Series accession number GSE12108 (http://www.ncbi.nlm.nih.gov/geo/query/acc.cgiaccGSE12108).

### Cell stimulation, lysis, Western blotting and ELISAs

Uninfected and infected cells were lysed in TN1 buffer (50 mM Tris (pH 8.0), 10 mM EDTA, 10 mM Na4P2O7, 10 mM NaF, 1% Triton X-100, 125 mM NaCl, 10 mM Na3VO4, 10 g/ml each aprotinin and leupeptin). Postnuclear lysates were boiled in Laemmli sample buffer and separated by SDS-PAGE, transferred to nitrocellulose filters, probed with the antibody of interest, then developed by ECL (Amersham Biosciences). For *F. tularensis* Schu S4 infections, cell pellets were boiled in Laemmli sample buffer as previously described [8]. Supernatants from Schu S4-infected cells were centrifuged (100 × g and then 10,000 × g), then passed through 0.22 µm filters and samples plated to ensure that there were no viable bacteria. ELISAs were done using sandwich ELISA kits from R & D Systems (IL-8) and eBioscience (IL-6, IL-1β).

### Flow cytometry

Cells were blocked with human IgG (Jackson ImmunoResearch, West Grove, PA, USA) in PBS for 10 minutes, then incubated with either PE-Cy7-conjugated anti-human CD14 antibody or isotype control (BD Pharmingen) for 60 minutes. In parallel, cells treated with only the FITC-conjugated goat anti-mouse antibody were prepared. Cells were then gently centrifuged, washed in PBS, and then resuspended in PBS containing 1% paraformaldehyde. Samples were then run on a BD FACSCalibur machine (Becton Dickinson).

### Real-time PCR

Quantitative reverse-transcription PCR was performed as described previously [13]. Briefly, RNA was isolated from human PBM using Trizol (Invitrogen, Carlsbad, CA), subjected to reverse transcription and then amplified using SYBR Green PCR master mix (Eurogentec North America, San Diego, Ca). Relative Copy Number (“RCN”) was calculated as described previously [13].

## Results and Discussion

### Global transcriptional responses to F. novicida and Schu S4

Human peripheral blood monocytes were isolated from four buffy coats and infected with the *F. tularensis tularensis* Schu S4 (“S4”) strain at 100 MOI for 24 hours. The 24 hour time point was chosen to match our previous microarrays performed with *F. novicida*-infected monocytes. These samples were then subjected to Affymetrix microarray analysis and combined with .CEL files generated earlier from *F. tularensis novicida* (“FN”) infection [8] for preprocessing and analysis. In total, 13512 transcripts were counted as significantly different with a fold difference of 2 or more.

**Figure 1:**
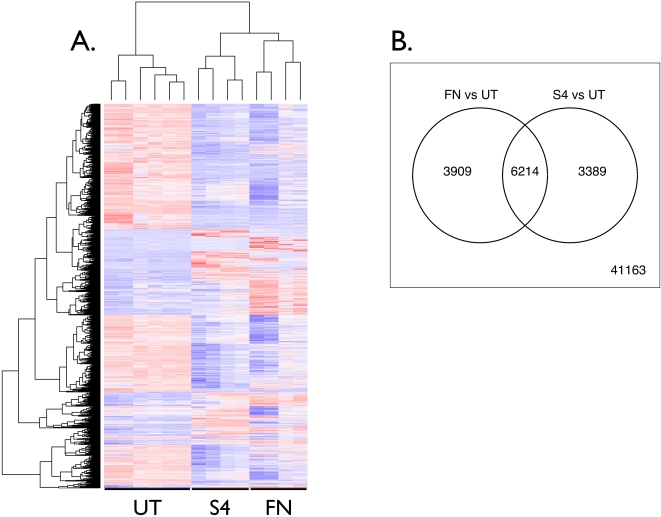
Transcriptional responses to *F. novicida* and *F. tularensis* Schu S4. A. Genes significantly different with an absolute fold change of 2 or greater after *F. novicida* or Schu S4 infection were pooled and used to create a heatmap. B. A Venn diagram of all significantly different genes that were up- or down-regulated twofold or more, with 6214 genes common to both strains, 3909 unique to FN, 3389 unique to S4 and 41163 counted as not different. FN: *F. novicida* (n = 4). S4: Schu S4 (n = 4). UT: untreated (n = 6).

One of the major goals was to compare transcriptional responses to the virulent Schu S4 versus the less virulent *F. novicida*. To acquire a general overview of these responses, these 13512 transcripts were plotted in the form of a heatmap [22] (Figure 1A). This shows great similarity in the responses to Schu S4 and *F. novicida*, agreeing with earlier findings that *F. novicida* shares a similar intracellular lifecycle with that of virulent *Francisella* (see [23] for review). Post-infection measurement of cell death was not feasible, but separate tests indicate that compared to untreated, up to 50% and 20% of the cells may have been dead following Schu S4 and *F. novicida* infection, respectively. Another test with a different donor showed no differences, however. All array chips were normalized together, mitigating the effect of possible differences in cell viability.

To quantify the overlap in response, a Venn diagram was created from the 13512 regulated transcripts (Figure 1B). Of these, 29% were unique to the *F. novicida* versus uninfected (“UT”), 46% were shared with both strains versus UT, and 25% were unique to S4 versus UT. The relatively large number of response genes unique to *F. novicida* or Schu S4 raised the possibility that some of these differences may help at least in part explain the high virulence of Schu S4. Hence, we examined several key immune response pathway genes in greater detail with the goal of gaining greater insight into the host cell subversion seen with *Francisella*. Significantly different genes were gathered and plotted as a heatmap (Figure S1). As demonstrated by this Figure, several genes responded differently to the two strains of *Francisella*. Below we study these in greater detail.

### Down-regulation of interferon pathway genes

**Figure 2:**
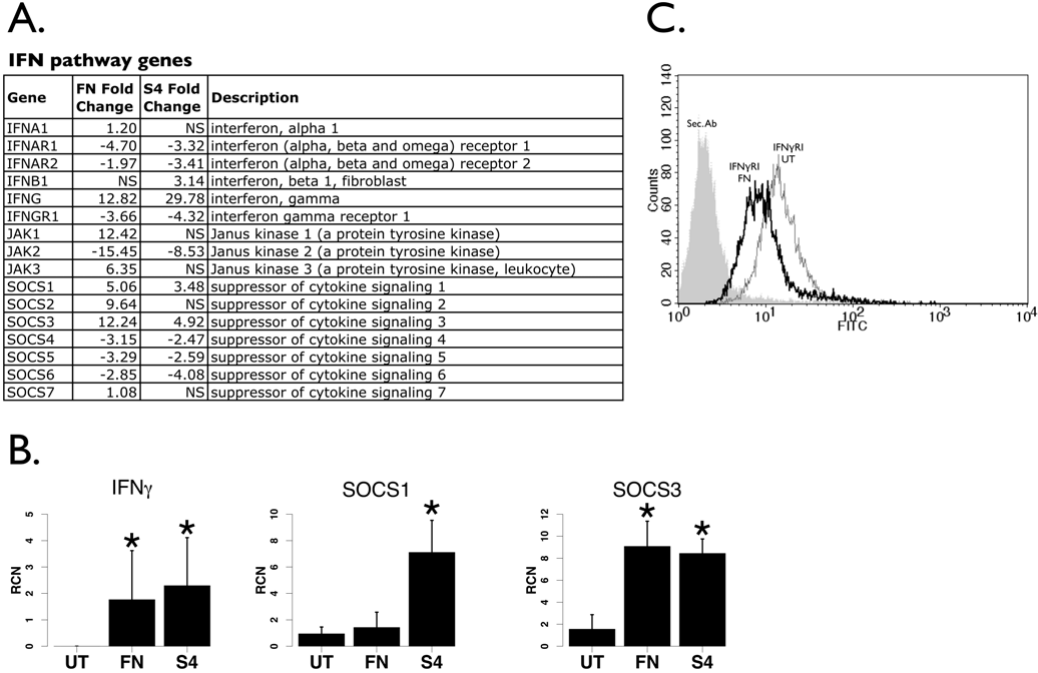
*Francisella*-induced changes in IFNγ pathway-related genes. A. List of IFN, Jak and SOCS genes regulated by *Francisella* infection (both *F. novicida* and Schu S4) from the microarray analysis. Fold changes of genes not statistically different are denoted “NS”. B. Real-time PCR of IFNγ, SOCS1 and SOCS3 from human PBM infected with either *F. novicida* (“FN”) or Schu S4 (“S4”) at 100 MOI for 24 hours (n = 8). “RCN” is Relative Copy Number for the Y axis. Asterisks denote statistical significance (p≤0.05) versus uninfected. C. Flow cytometry for the IFNγ receptor following *F. novicida* infection of human monocytes. The data are representative of three independent experiments.

In our initial analysis of infected monocytes [8] we found an up-regulation of IFNγ, a cytokine shown to confer host protection in murine models [24], [25], [26] and found after infections in humans and mice [27], [28], [29], although the strain of mice may be important [30] as well as route of infection [31]. IFNγ has been shown to antagonize phagosomal escape [32], [33], leading to improved host cell protection. It has also been reported that Type I interferon is induced following a cytosolic sensing event and plays a role in the activation of the inflammasome during *F. novicida* infection in mice [34]. Activation of the inflammasome is critical for the post-translational processing and release of IL-1β [13], [14]. Hence, we examined the expression of interferon-related genes (Figure 2A). We found that the IFNα receptor was down-regulated following both *F. novicida* and Schu S4 infection, with a concomitant induction of ligand expression with *F. novicida* but not with Schu S4. IFNβ was up-regulated after Schu S4 but not *F. novicida* infection. As previously reported [8], IFNγ was up-regulated, but Schu S4 elicited more than twice the response as *F. novicida*. However, the IFNγ receptor was down in both cases, suggesting that IFNγ may elicit a suboptimal response after both types of infections.

Jak proteins act downstream of IFN signaling, and we found that Schu S4 caused a down-regulation in Jak2 with no change in Jak1 nor Jak3. There was strong up-regulation of both Jak1 and Jak3 and down-regulation of Jak2 after *F. novicida* infection, however. The Suppressor of Cytokine Signaling 1 and 3 (SOCS1 and SOCS3) genes, negative regulators of IFNγ-induced Jak/STAT1 signaling [35] were up-regulated by both bacterial strains, although a twofold greater SOCS3 than SOCS1 induction was seen with *F. novicida* (Figure 2A).

Real-time PCR analysis was performed to verify IFNγ, SOCS1 and SOCS3 responses (Figure 2B), and results confirmed up-regulation of these genes, although significant up-regulation of SOCS1 following *F. novicida* infection could not be verified. The IFNγ receptor was tested using flow cytometry after *F. novicida* infection, and results showed clear down-regulation of this receptor (Figure 2C). Collectively, these data indicate that there is an overall suppression of the interferon signaling pathway, both Type I and Type II, and suggest that the infected monocytes, even if exposed to exogenous IFNα or IFNγ, would exhibit a suboptimal response. Indeed, in a recent study we have demonstrated that *F. novicida* infection dampens the ability of murine macrophages to respond optimally to IFNγ, resulting in reduced nitric oxide production and greater intracellular bacterial growth [54].

### Toll-like receptor signaling is compromised

**Figure 3:**
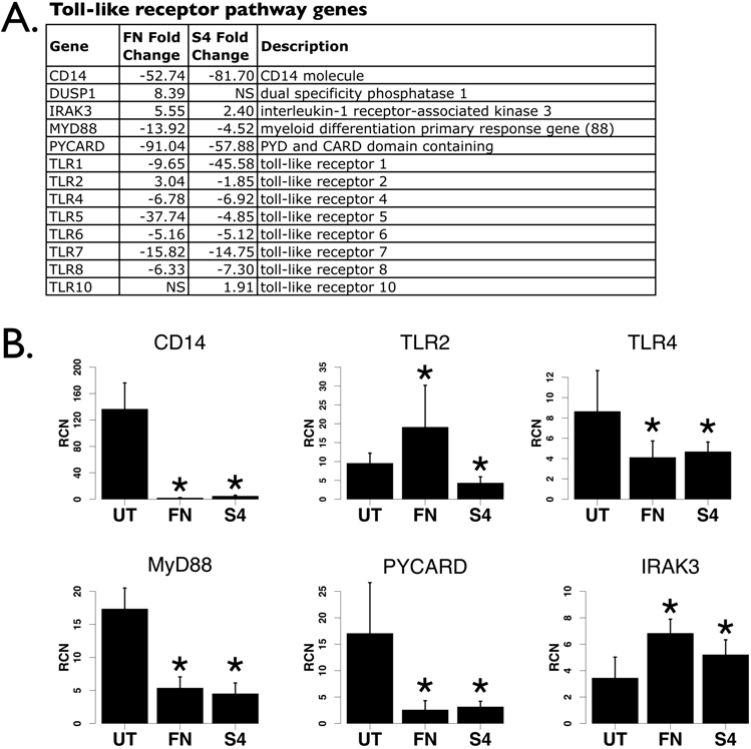
Toll-like receptor pathway genes affected by *Francisella*. A. List of TLR pathway genes from the microarray analysis. Fold changes of genes not statistically different are denoted “NS”. B. Real-time PCR analyses of CD14, TLR2, TLR4, MyD88, PYCARD and IRAK-M from human PBM infected with either *F. novicida* (“FN”) or Schu S4 (“S4”) at an MOI of 100 for 24 hours (n = 8). “RCN” is Relative Copy Number for the Y axis. Asterisks denote statistical significance (p≤0.05) versus uninfected.

**Figure 4:**
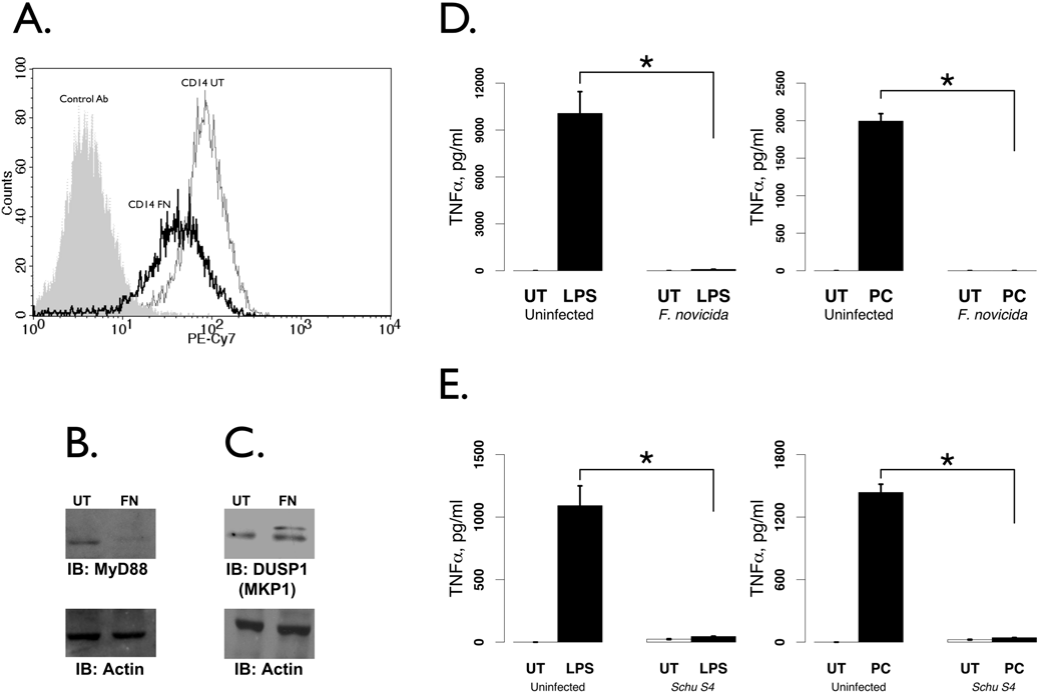
Hyporesponsiveness of TLR signaling after *Francisella* infection. A. Flow cytometry of CD14 in infected versus uninfected monocytes, gating on live cells by scatter. B and C. Western blot of MyD88 (B) and MKP1 / DUSP1 (C) after *F. novicida* infection of THP-1 cells. Actin reprobes (bottom panels) show equal loading. D and E. THP-1 cells were first infected with *F. novicida* (D) or Schu S4 (E) for 14 hours or left uninfected. Uninfected and infected cells were then treated with gentamicin at 50 µg/ml to kill extracellular bacteria and restimulated with LPS (500 ng/ml, D and E left panels) or Pam3CSK (100 ng/ml, D and E right panels) for 8 hours. Cell supernatants were analyzed for TNFα by ELISA. Values from three independent infections were analyzed by student's t-test. Asterisks denote statistical significance (p≤0.05).

Toll-like receptor signaling is responsible for sensing pathogens and prompting cellular immune responses. Upon contact with the host cell, *Francisella* elicits signaling through Toll-like Receptor 2 (TLR2) [36], [37]; the lipopolysaccharide of *Francisella* elicits only very weak TLR4 signaling [38], [39] that depends on the co-receptor CD14 for greatest activity [40]. We found that TLR1,4,5,6,7 and 8 were all down-regulated following infection with either *F. novicida* or Schu S4 (Figure 3A). TLR2, however, was up-regulated with *F. novicida* infection as previously reported for *F. tularensis* Live Vaccine Strain [41] yet markedly down-regulated with Schu S4 (Figure 3A). Regulation of TLR2, TLR4, CD14, MyD88, PYCARD and IRAK-M were verified by real-time PCR analysis (Figure 3B). As CD14 interacts with both TLR4 [42] and TLR2 [43], [44], the data suggest that signaling through either TLR pathway would be suboptimal. Flow cytometry using *F. novicida*-infected monocytes showed a strong down-regulation of CD14 (Figure 4A). Similarly, the downstream mediator MyD88 [45], [46] was decreased at the protein level (Figure 4B). The inflammasome protein PYCARD (ASC) [47] was reduced following infection, while the negative regulator IRAK-3 (IRAK-M) was increased (Figure 3B). Further, a negative regulator of TLR pathway activity, MKP1 (DUSP1) [48], was increased (Figure 4C), although the 3.69-fold increase with Schu S4 did not reach statistical significance (p = 0.057, Figure 3A). This overall pattern of regulation suggests that after infection, TLR signaling would be compromised.

Consistent with this, we found that the monocytic cell line THP-1 showed significant hyporesponsiveness to TLR4 (Figure 4D, left panel) and TLR2 (Figure 4D, right panel) stimuli following *F. novicida* infection, as measured by TNFα ELISA. The same experiment using Schu S4 led to similar results, with THP-1 cells being hyporesponsive to TLR4 (Figure 4E, left panel) and TLR2 (Figure 4E, right panel). These results agree closely with studies in the murine system that showed hyporesponsiveness to TLR stimulation after *Francisella* infection [49], [50].

Although TLR2 expression was increased after infection with *F. novicida*, the down-regulation of CD14 and MyD88 along with up-regulation of MKP1 and IRAK-3 offer a molecular mechanism for why TLR responses are dampened following *Francisella* infection. Up-regulation of the negative regulator MKP1 was not found to be statistically significant after Schu S4 infection, but the expression of TLR2 itself was significantly down-regulated. Hence, although hyporesponsiveness was demonstrated following infection with either strain, the TLR2 pathway, responsible for inflammatory responses to *Francisella* [36], [37], may be specifically compromised by the virulent strain.

### Schu S4 down-regulates the PI3K / Akt pathway and inflammatory cytokine production

**Figure 5:**
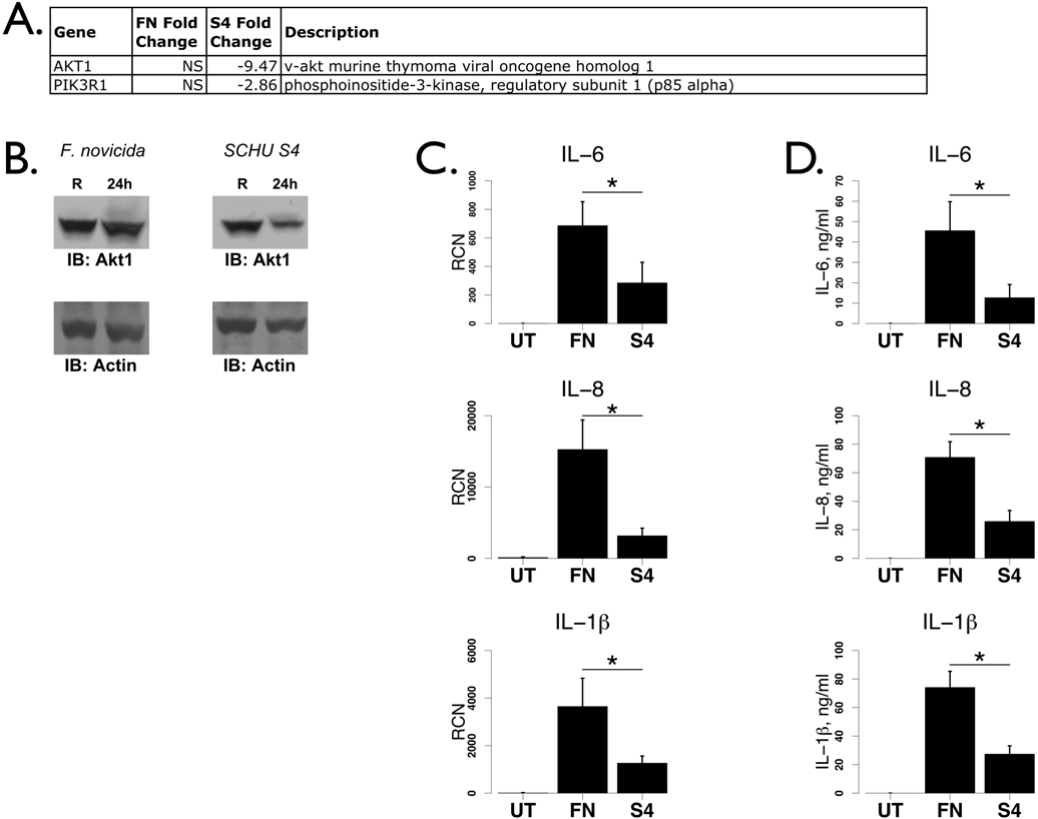
Dampened cytokine response in Schu S4-infected monocytes. A. Table of PI3K and Akt response genes from the microarray analysis. Genes not significantly different are denoted with “NS” in the Fold Change columns. B. Western blots for Akt1 after *F. novicida* (left) and Schu S4 (right) infection of human monocytes. Actin reprobes showed equivalent loading (bottom panels). The data are representative of three independent observations. C. Real-time PCR analysis of IL-8, IL-6 and IL-1β transcripts after infection of human peripheral blood moncytes by *F. novicida* (“FN”) and Schu S4 (“S4”) (n = 8). “RCN” is Relative Copy Number for the Y axis. D. ELISAs showing IL-8, IL-6 and IL-1β after infection by *F. novicida* and Schu S4 in human monocytes (n = 4). Data were analyzed by student's t-test. Asterisks denote statistical significance (p≤0.05).

We recently demonstrated that activation of the PI3K/Akt pathway is critical for the activation of NFκB and the subsequent pro-inflammatory gene transcription during *F. novicida* infection [16]. Thus, we examined the regulation of this pathway by first examining the expression of genes in the phosphoinositol and protein kinase B (Akt) ontology groups. We plotted the average expression values for genes significantly different in the untreated versus *F. novicida*, untreated versus Schu S4 or *F. novicida* versus Schu S4 comparisons (Figure S1). Compared to untreated, infection with both strains led to substantial down-regulation of these pathway members. In addition, more genes were down-regulated after Schu S4 infection compared to *F. novicida* such as PIK3R1 (p85 subunit of PI3K) and Akt1 (Figure S1 and Figure 5A). These results suggest that both strains dampen PI3K/Akt signaling, but Schu S4 elicits a stronger dampening. We then tested the expression of Akt1 at the protein level since it has been shown to be critical for cytokine responses [16], [17]. As expected, Western blotting of lysates from human monocytes infected with either *F. novicida* or Schu S4 indicated a strong decrease at the protein level in Schu S4-infected cells but not in *F. novicida*-infected cells (Figure 5B). This, especially in combination with the reduced TLR2, predicts that cytokine responses would be lower with Schu S4 than with *F. novicida* infection.

Consistent with reduced TLR2 and PI3K/Akt, real-time PCR analysis showed that although IL-8, IL-6 and IL-1β were induced by both bacterial strains, Schu S4 elicited significantly less transcript than *F. novicida* (Figure 5C). Protein levels were also significantly lower with Schu S4 as measured by ELISAs (Figure 5D). Hence, it appears that the Schu S4 strain of *Francisella* elicits a significantly lower pro-inflammatory response than the less virulent subspecies. The suppressive effect of Schu S4 on members of the PI3K and Toll-like receptor pathways, both necessary for *Francisella*-induced NFκB-dependent gene transcription, represents a fundamental weakness in the host inflammatory response to *Francisella* infection.

In examining genes involved in other host defense mechanisms, we found that both strains of *Francisella* led to decreases in MHC Class II genes (Figure S2, panel A). Similarly, genes involved with autophagy, a process which can also contribute to antigen presentation [51], were down-regulated (Figure S2, panel B). *Francisella* has been shown to re-enter the endocytic pathway via autophagy following phagosomal escape and cytoplasmic replication, and this can be reversibly blocked by chloramphenicol treatment [52]. Perhaps the down-regulation in autophagy-related genes reflects control by *Francisella* during its replication phase in the cytoplasm as suggested by Cheeroun *et al*.

In addition, phagocytosis-related genes were decreased including Fcγ receptors, the mannose receptor, complement components and receptors, scavenger receptors [53] and the phagocytic pathway-related signaling components Fyn and Syk [54] (Figure S3). There were some differences, however, between *F. novicida* and Schu S4 with regard to regulation of various phagocytic pathway members. Fyn and Scavenger Receptor F1 were up-regulated in only *F. novicida*-infected monocytes, and FcγRIIa was significantly decreased in only Schu S4-infected monocytes. Collectively, these data suggest that *Francisella* infection decreases the ability of monocytes to ingest subsequent particles or pathogens.

Taken together, these results point to several subversive mechanisms that may help explain the virulence of *F. tularensis* Schu S4 and reinforce its reputation as a stealth pathogen. The quantitative differences in cytokine response may be attributable to the qualitative differences seen, especially with p85 and Akt1 expression. The nature of these qualitative differences suggests that Schu S4 actively suppresses, rather than simply bypasses, the host innate immune response.

Although several categories of immune response genes were affected, augmenting the PI3K/Akt1 pathway could be examined as a possible means to combat *Francisella* infection, especially in the event that antibiotic-resistant strains become commonplace. Indeed, in mice it has been shown that overactive Akt1 confers a survival advantage [17]. *Francisella tularensis* Schu S4 down-regulates the PI3K and Akt1 genes, so identifying the mechanism(s) by which this happens and / or examining events downstream of Akt1 that are responsible for the survival advantage may lead to specific ways to combat tularemia, perhaps as an augmentation to standard antibiotic treatment.

It must also be considered that, as shown by Andersson et al. [55] the transcriptional responses to *Francisella* are often dynamic. Future transcriptional analyses on human monocytes could be done using different timepoints. Finally, an examination of isolated cells versus cocultured cells may yield more insights, as intercellular communication can affect cellular responses.

## Supporting Information

Figure S1 Genes with ontology entries of “phosphoinositol,” “phosphoinositide,” “protein kinase B,” “antigen,” “autophagy,” “interferon,” “Toll,” “phagocytosis,” “JAK-STAT” or “cytokine” that were significantly different in untreated versus *F. novicida* or untreated versus Schu S4 comparisons with a fold difference of at least 3 were chosen for the plot. Highlighted in yellow are genes verified by either real-time PCR or Western blotting. Blue indicates low expression and red high expression. Row-by-row scaling was done for the color mapping.
(2.24 MB TIF)

Figure S2 Genes in antigen presentation and autophagy, from the microarray analysis. An “NS” in the Fold Change column denotes a non-significant change. A. Table of genes involved in antigen presentation. B. Table of autophagy-related genes.
(1.03 MB TIF)

Figure S3 Genes involved in phagocytosis, from the microarray analysis. An “NS” in the Fold Change column denotes a non-significant change.
(1.26 MB TIF)
