# Supplementary figures and images for: Microarray Analysis of Human Monocytes Infected with Francisella tularensis Identifies New Targets of Host Response Subversion
Source: PLoS One. 2008 Aug 13;3(8):e2924. doi: 10.1371/journal.pone.0002924 (PMC2488368; doi:10.1371/journal.pone.0002924)

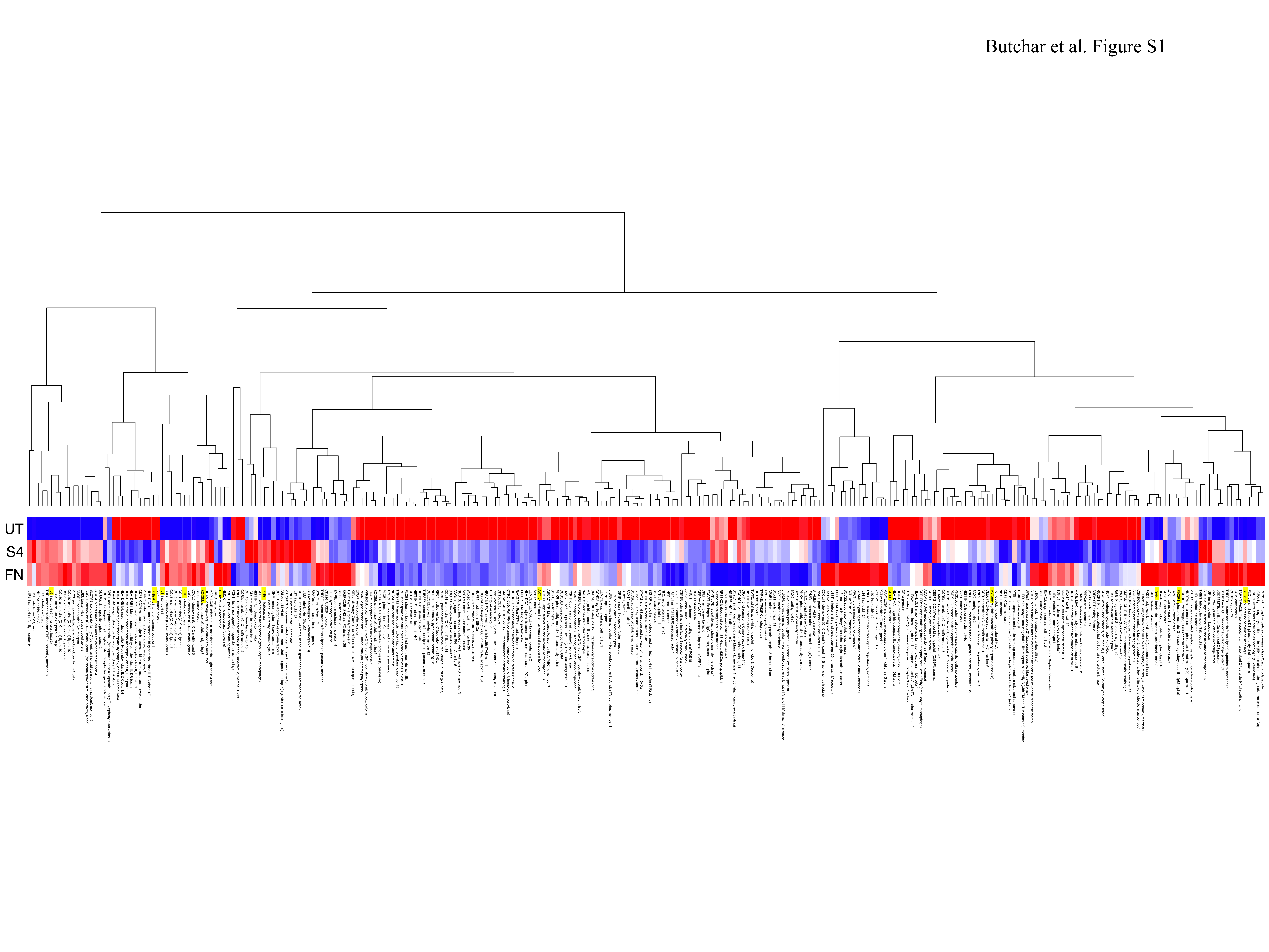

Supplement: Figure S1 — Genes with ontology entries of “phosphoinositol,” “phosphoinositide,” “protein kinase B,” “antigen,” “autophagy,” “interferon,” “Toll,” “phagocytosis,” “JAK-STAT” or “cytokine” that were significantly different in untreated versus F. novicida or untreated versus Schu S4 comparisons with a fold difference of at least 3 were chosen for the plot. Highlighted in yellow are genes verified by either real-time PCR or Western blotting. Blue indicates low expression and red high expression. Row-by-row scaling was done for the color mapping. (2.24 MB TIF) [file pone.0002924.s001.tif]

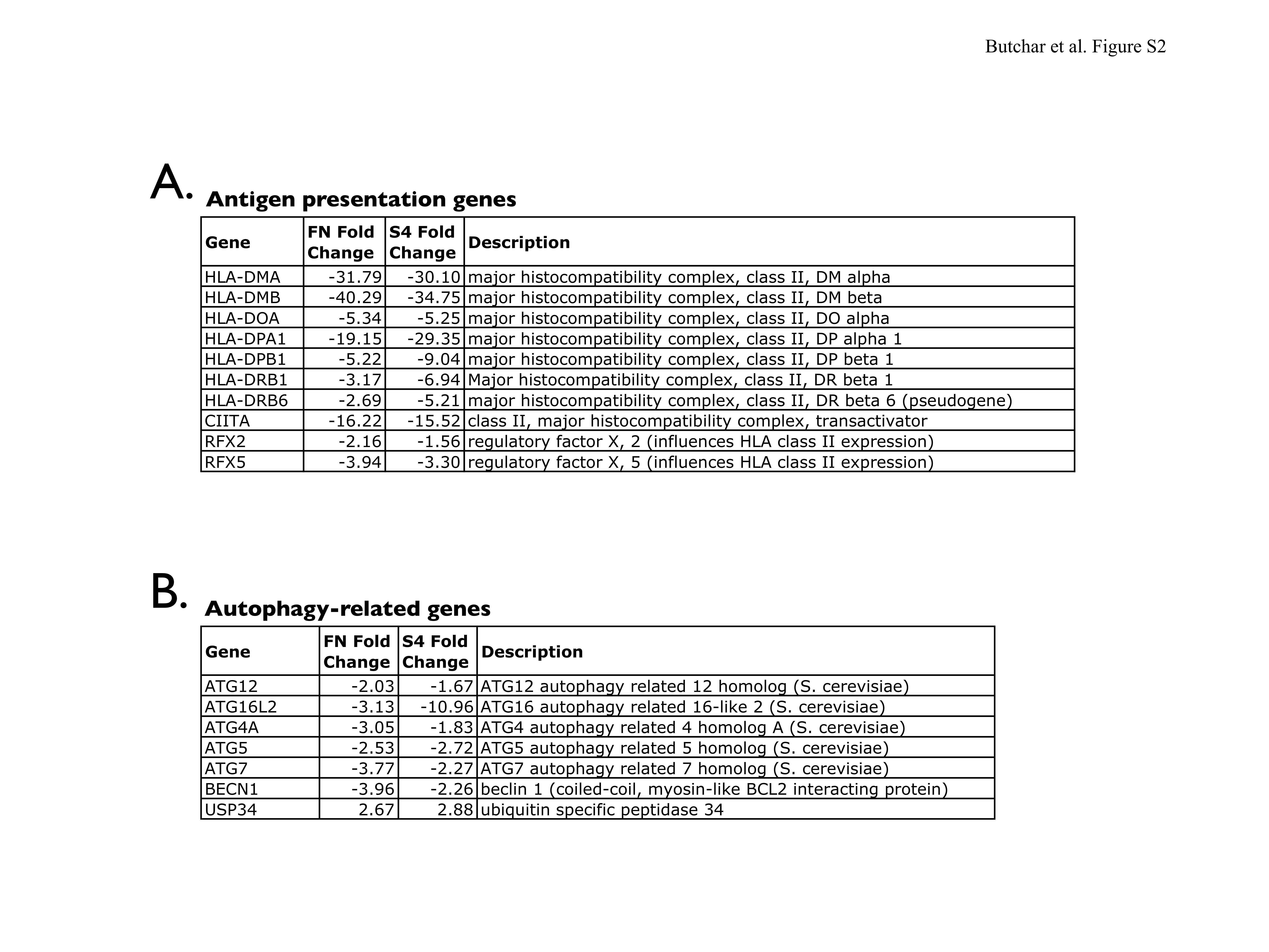

Supplement: Figure S2 — Genes in antigen presentation and autophagy, from the microarray analysis. An “NS” in the Fold Change column denotes a non-significant change. A. Table of genes involved in antigen presentation. B. Table of autophagy-related genes. (1.03 MB TIF) [file pone.0002924.s002.tif]

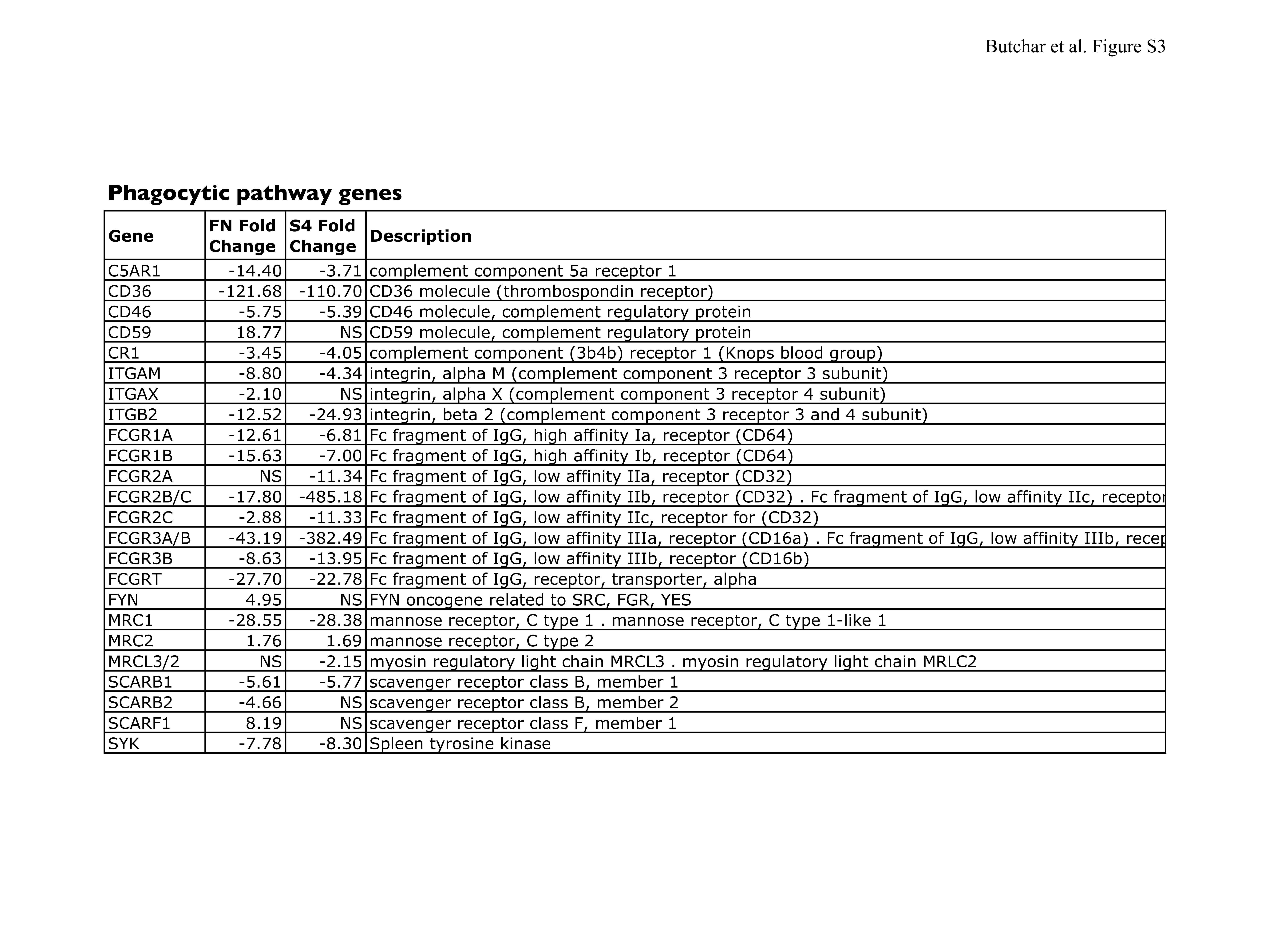

Supplement: Figure S3 — Genes involved in phagocytosis, from the microarray analysis. An “NS” in the Fold Change column denotes a non-significant change. (1.26 MB TIF) [file pone.0002924.s003.tif]
